# Effect of Low‐Carbohydrate Diets on C‐Reactive Protein Level in Adults: A Systematic Review and Meta‐Analysis of Randomized Controlled Trials

**DOI:** 10.1002/fsn3.70566

**Published:** 2025-07-18

**Authors:** Mahdieh Khodarahmi, Hooria Seyedhosseini, Gholamreza Askari

**Affiliations:** ^1^ Nutrition and Food Security Research Center, School of Nutrition and Food Science Isfahan University of Medical Sciences Isfahan Iran

**Keywords:** carbohydrate restriction, C‐reactive protein, inflammation, low‐carbohydrate diet, meta‐analysis

## Abstract

Chronic low‐grade inflammation is a probable mediator between the quantity of carbohydrate intake and health outcomes. The aim of this systematic review and meta‐analysis of randomized clinical trials (RCTs) was to summarize the effects of low carbohydrate diets (LCDs) as compared to control diets on c‐reactive protein (CRP) levels in adults. A comprehensive search was conducted in several databases up to February 2024. In total, 60 eligible trials with 5511 adults were included in the meta‐analysis. LCDs resulted in an average reduction of 0.18 mg/L in CRP levels compared to control groups (MD = 0.18; 95% CI: 0.032 to 0.03; *p* = 0.016); however, sensitivity analysis revealed that this significance was dependent on a single study (Abbaspour Rad et al.), with exclusion of which the effect became non‐significant (MD = −0.14; 95% CI: −0.28 to 0.00; *p* = 0.052), indicating the fragility of the overall finding. Based on subgroup analyses, LCDs achieved greater lower CRP levels in trials of long‐term duration (> 12.5 weeks) (MD = −0.24; 95% CI: −0.48 to −0.00; *p* = 0.046), in younger people (≤ 49.6 years) (MD = −0.30; 95% CI: −0.51 to −0.10; *p* = 0.003) with baseline CRP concentrations of more than 4.5 mg/L (MD = −0.70; 95% CI: −1.13 to −0.26; *p* = 0.002), and in obese participants with BMI > 35 (MD = −1.21; 95% CI: −1.91 to −0.51; *p* = 0.001). Also, according to multivariate meta‐regression analyses, baseline CRP level remained a strong predictor of the treatment effect of LCDs (*p* = 0.017). Evidence from our study showed that LCDs may modestly improve CRP levels in adults. However, the effect appears more pronounced in individuals with higher baseline CRP levels, greater BMI, and younger age, indicating that these factors may modify treatment response. Future well‐designed RCTs are required, with particular attention to dietary confounders such as protein and fat sources and carbohydrate quality.

## Graphical abstract

Chronic low‐grade inflammation is a probable mediator between quantity of carbohydrate intake and health outcomes. The results of the present systematic review and meta‐analysis showed that low carbohydrate diets are an effective dietary strategy for improving CRP levels especially among obese adults with baseline CRP concentration more than 4.5 mg/L.

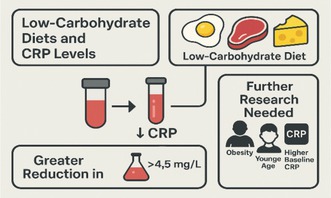

## 1 Introduction

Inflammation acts as a double‐edged sword: while it is a key immune defense mechanism against injury and infection, chronic low‐grade inflammation is a common pathological characteristic of a wide variety of chronic outcomes, namely, type 2 diabetes mellitus (T2DM), cardiovascular diseases (CVDs), metabolic syndrome (MetS), non‐alcoholic fatty liver disease (NAFLD), chronic kidney disease, various cancers, and neurodegenerative disorders (Furman et al. 2019).

C‐reactive protein (CRP), a prominent and frequently studied marker of inflammation, is involved not only in cardiovascular risk prediction but also in the pathogenesis of a wide range of health conditions. In this context, high CRP concentrations have been associated with adverse clinical consequences in various conditions, such as T2DM (Erkus et al. 1992), diabetic nephropathy (Aktas 2024; Bilgin et al. 2021), autoimmune thyroiditis (Demirkol and Aktas 2022), chronic hepatitis (Demirkol et al. 1992), and even COVID‐19 infection (Demirkol et al. 2022). As well as, CRP is gaining recognition as a prognostic indicator in the management of critically ill patients, especially those in intensive care units (Aktas et al. 2024). Considering its strong relationship with multiple inflammatory pathologies, CRP is regarded as a useful and modifiable biomarker that may be utilized for both preventive and therapeutic strategies (Danesh et al. 2004) (Beyhoff et al. 2020). Consequently, finding an effective way to reduce CRP levels may improve patient health outcomes.

Importantly, diet has been indicated to be a potential moderator of chronic inflammation, and lifestyle modification like dietary change can exert therapeutic effects (Wang et al. 2020; Khodarahmi et al. 2019). An extensive body of evidence has suggested that disparities in dietary macronutrient composition, particularly carbohydrate and fat intake, may attenuate disease risk (Krieger et al. 2006; Mansoor et al. 2016; Barber et al. 2021). Low‐carbohydrate diets (LCDs), in which carbohydrates (Floegel and Pischon 2012) are restricted and replaced by further consumption of fat and/or protein, are gaining substantial popularity as a beneficial strategy for protection against many dysfunctions (Barber et al. 2021; Mooradian 2020). Specifically, emerging evidence indicates that LCDs may attenuate systemic inflammation by enhancing insulin sensitivity, decreasing adiposity, and modulating lipid metabolism, with additional findings highlighting their potential to ameliorate inflammation‐related processes such as cardiovascular risk factors and obesity‐related metabolic disturbances (Samaha et al. 2003; Goldenberg et al. 2021; Minihane et al. 2015).

There are heterogeneities in LCD definition, but an LCD is typically defined as a diet that restricts carbohydrate intake to below 26% or 130 g/day (Macedo et al. 2020; Landry et al. 2021). Other categories of LCDs include moderate LCDs (26%–44% of total energy or 130–225 g/day) and very low carbohydrate diets (≤ 10% of total energy or 20–50 g/day) (Landry et al. 2021). Evidently, according to the recommendation of The Institute of Medicine, people should normally consume between 45% and 65% of total daily energy from carbohydrate, and this accordingly implies that carbohydrate intake less than 45% can be regarded as an LCD (Trumbo et al. 2002). However, due to LCDs limiting carbohydrate in favor of liberal intake of protein and fats, especially saturated fatty acids (Jenkins et al. 2014), there are some concerns about their detrimental influences on CVD risk factors, in particular CRP (Floegel and Pischon 2012; Santos et al. 2012). On the other hand, diets with high levels of carbohydrates often comprise refined grains with high glycemic index, stripped of fiber and other important nutrients (vitamins, antioxidants and so on) may promote inflammation and oxidative stress (Ghorbani et al. 2023; Buyken et al. 2014). Undoubtedly, greater precise knowledge is needed to elucidate the contribution of LCDs to inflammatory markers.

Effectiveness of LCDs in reducing inflammation has been shown in some previous studies. In this regard, more recently, a robust review of a human studies concluded that LCDs are able to decrease inflammation (Field et al. 2023). Nevertheless, evidence from another meta‐analysis in patients with T2DM did not show any improvements in CRP levels following an LCD compared to a low‐fat diet (LFD) (Apekey et al. 2022). In contrast, a meta‐analysis of 44 trials published to March 2022 reported favorable effects of LCDs on CRP concentration in adults (Kazeminasab et al. 2024). Nonetheless, it is not well determined whether the association of LCDs with CRP levels is dependent on baseline inflammatory condition. Besides, the effects of LCDs on this inflammatory marker according to the quality of dietary fats are still unclear. Additionally, several newly published RCT studies with conflicting data in this area are available, which highlights the need for re‐evaluation of this topic with a higher statistical power in a new extensive review.

Considering the lack of consensus across prior studies, the current systematic review and meta‐analysis of randomized controlled trials (RCTs) was conducted to summarize and quantify the effects of LCDs on CRP levels to attain a more explicit outcome.

## 2 Materials and Methods

### 2.1 Literature Search

The recommendations of the Preferred Reporting Items for Systematic reviews and Meta‐Analysis (PRISMA) framework were followed for performing the present study (Moher et al. 2009). The protocol of this systematic review and meta‐analysis has been registered and publicized in the International Prospective Register of Systematic Reviews (PROSPERO, registration no: CRD42023387452). It should be noted that the aim of this research was to summarize the inconsistent findings from RCTs to draw a high‐quality conclusion with regard to the influence of LCDs on inflammatory factors (CRP, TNF‐a, IL‐6 and adhesion molecules). Nonetheless, due to the considerable number of identified studies and the massive volume of the results, the current study was limited to the published data concerning the impact of LCDs on CRP concentration. Hence, all keywords related to inflammation were included in the primary search. The main electronic databases, including PubMed, EMBASE, Web of Science, and Scopus, were searched for relevant RCTs until February 2024, without any restrictions on time and language, using the Medical Subject Headings (MeSH) and/or text words, which are shown in detail in Supplementary File S1. The search strategy also contained keywords related to serum glucose, lipid profile, and thrombosis factors, as inflammatory markers might be reported as secondary endpoints in some trials. Additionally, the reference lists of the selected original studies and review articles were manually inspected in order to avoid missing any relevant citations.

### 2.2 Eligibility Criteria and Study Selection

Two independent reviewers (M.K. and H.S.) screened retrieved relevant records to determine eligibility, and any disagreements were resolved by an additional reviewer (G.A.). Manuscripts were selected to be included in this review if they fulfilled the following criteria: (1) RCTs published in English language; (2) investigated the effects of LCDs (< 45% of total energy from carbohydrates) on CRP levels among adults (≥ 18 years) in spite of their health condition; (3) reported data on changes of CRP across study arms as an outcome or enough information to calculate this estimate. Accordingly, studies were excluded if they: (1) did not have a control group or comparison arm; (2) were lacking net change values of the outcomes of interest or required data to compute them; (3) assessed the impacts of LCDs alongside other dietary exposures simultaneously, and it was impossible to examine the influence of LCDs alone; (4) reported other inflammatory factors as their outcomes instead of CRP. Since adaptation to a new diet usually lasts 14 days, short‐term trials (< 2 weeks) were ignored for inclusion in analyses as well.

### 2.3 Data Extraction and Quality Assessment

Both processes of data extraction and methodological quality appraisal of included trials were carried out independently by two authors (M.K. and H.S.) and conflicting judgments were resolved through consulting the third author (G.A.). Information sought for extraction was as follows: first author's last name, date of publication, country, demographic and anthropometric characteristics of subjects (age range or mean age, gender, body mass index (BMI), health condition), study design (parallel/cross‐over), follow‐up length, sample sizes for both the intervention and control arms, dietary interventions implemented in the LCDs and control groups, and mean change values of the desired outcome and their corresponding standard deviations. If the results of a study were published in more than 1 article, the most recent paper with more complete information was included in analyses. When a study reported the results of multiple strata, each of them was considered a separate intervention.

The methodological quality of the included trials was assessed by the Cochrane Collaboration tool (Higgins et al. 2023) indicating the main types of bias in clinical trials as follows: selection bias (random sequence generation, allocation concealment); performance bias (blinding of participants and personnel and masking of the outcome assessors); attrition bias (incomplete outcome data) and reporting bias (selective result reporting). Overall judgment for each individual study was classified as ‘good’ quality if there was low risk of bias for ≥ 3 items, ‘fair’ if there was low risk for two items; and as ‘weak’ if there was low risk for < 2 items based on the guidelines provided by this instrument. All values of outcome of interest expressed in different units were converted to mg/L by proper conversion factors.

### 2.4 Data Synthesis and Statistical Analysis

The estimated effect of interest was the pooled mean differences and their corresponding standard deviations (McCullough et al. 2022) of CRP between intervention and control groups. For studies that did not provide SDs of the change, the following formula suggested by Follmann et al. was used to derive them: SD changes = square root [(SD baseline^2^ + SD final^2^) −(2 × *R* × SD baseline × SD final)] with a correlation coefficient of 0.5 (Follmann et al. 1992). For studies in which outcome variables were expressed as median and range (or 95% confidence interval (CI)), the methods described by Hozo and colleagues were applied (Hozo et al. 2005). Besides, in the case of reporting standard errors (SEs), SDs were calculated by multiplying SEs by the square root of the sample size in each group (Hozo et al. 2005).

Meta‐analyses were undertaken using the Stata software (version 17) to calculate the overall weighted mean differences (WMD) and its 95% CIs using a random effects model, based on the DerSimonian and Laird method (DerSimonian and Laird 1986). To assess the presence of heterogeneity, Cochrane's *Q*‐test was used and the extent of heterogeneity was quantified through *I* ^2^ statistic (Higgins and Thompson 2002). To find out probable sources of heterogeneity, subgroup analyses were conducted based on some important variables including: health condition (healthy, overweight and obese, T2D, MetS and cardiometabolic risk factors and cancers), participants' gender (male, female or both genders), mean age of subjects (≤ 49.6 or more), mean baseline CRP level (≤ 4.5 or more), follow‐up duration (≤ 12.5 weeks or more), mean baseline BMI (≤ 35 or more), proportion of carbohydrate from calorie (≤ 10% (20–50 g/d), 11%–26% (50–130 g/day) and 27%–44% (130–225 g/day)), trial total sample size (≤ 46 or more), methodological quality (good, fair and weak) and the quality of dietary fats (SFA ≤ 10% or more). To further detect the impacts of these variables on CRP concentration response to LCDs, univariate and multivariate meta‐regression analyses were also carried out. Moreover, we performed sensitivity analyses to examine the influence of an individual study on the overall effect size by the stepwise omission of one study at a time. The probability of potential publication bias was evaluated by inspection of funnel plot asymmetry (Egger et al. 1997), Begg's rank correlation, and Egger's weighted regression tests (*p* < 0.10) (Egger et al. 2001). Subsequently, when there was any significant publication bias, the trim‐and‐fill approach was used to provide an adjusted summary effect in the presence of publication bias (Duval and Tweedie 2000).

## 3 Results

**FIGURE 1:**
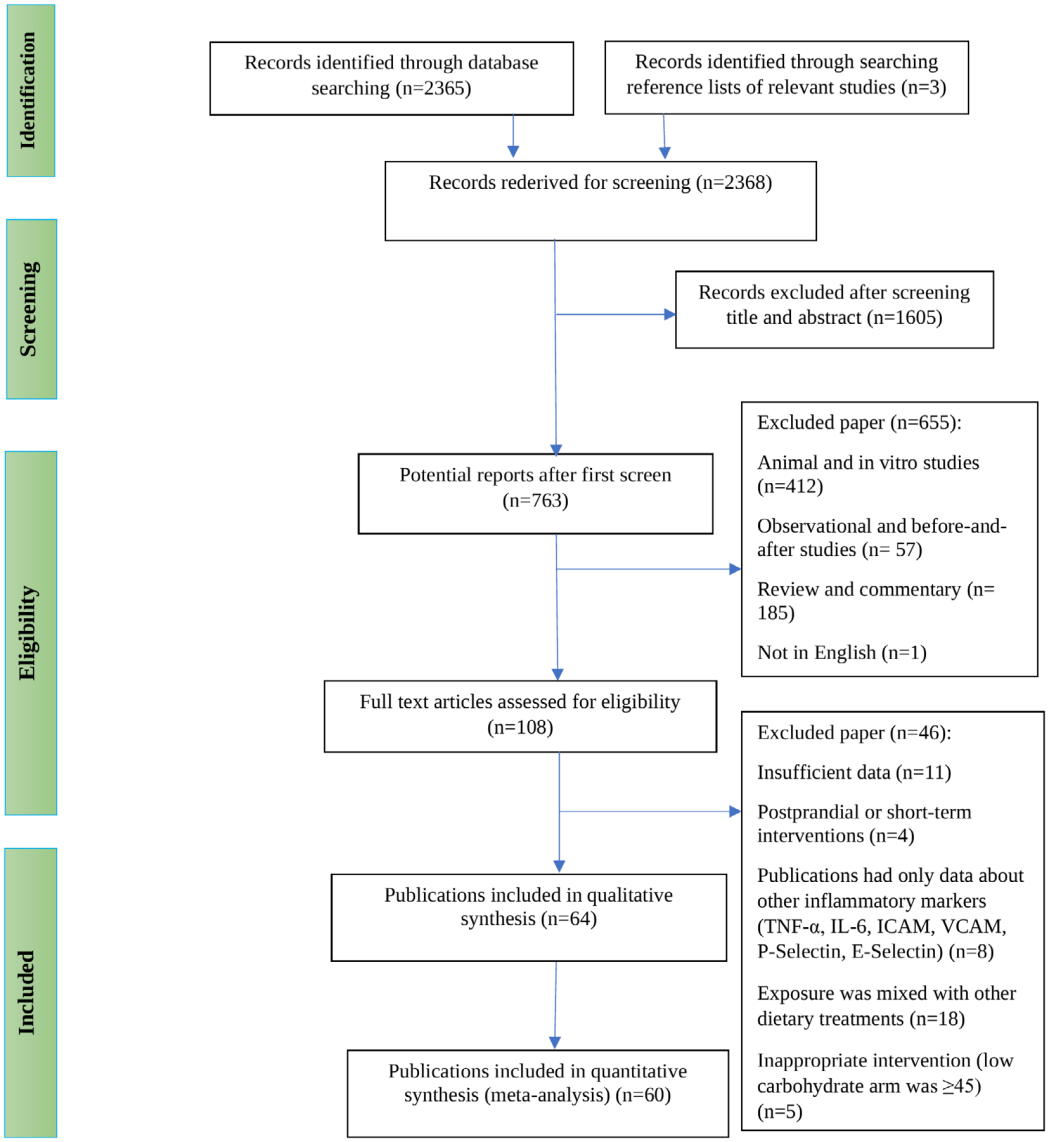
Flow diagram of study selection for meta‐analysis.

A total of 2368 relevant records were identified during the initial literature search, of which 2208 were excluded after reviewing the titles and abstracts for the reasons summarized in Figure 1. Thus, 108 studies remained for full‐text review; of those, 46 records were eliminated as they did not meet all of the eligibility criteria. The main reasons for exclusion were as follows: (a) eleven did not provide sufficient data to be included in the meta‐analysis, (b) four administered extremely short‐term interventions, (c) eight reported data for other inflammatory factors instead of CRP, and (d) eighteen prescribed LCD alongside other commonly used dietary regimens. Finally, out of 64 trials included in the present systematic review, 4 studies (McCullough et al. 2022; Gram‐Kampmann et al. 2023; Perissiou et al. 2020; Thomsen et al. 2022) were dropped since they had undetectable or very high baseline CRP concentrations. Hence, 60 references with 66 datasets were included in the quantitative meta‐analysis (Jenkins et al. 2014; Barbosa‐Yañez et al. 2018; Bhanpuri et al. 2018; Breukelman et al. 2021; Brinkworth et al. 2009; Brinkworth, Noakes, Keogh, et al. 2004; Brinkworth, Noakes, Parker, et al. 2004; Budipramana 2020; Cardillo et al. 2006; Dansinger et al. 2005; Davis et al. 2011; de Luis et al. 2007, 2015, 2020; Durrer et al. 2021; Ebbeling et al. 2022; Erdem et al. 2022; Flechtner‐Mors et al. 2010; Forsythe et al. 2008; Freedland et al. 2020; Goss et al. 2020; Gower and Goss 2015; Harvey et al. 2019; Johnston et al. 2006; Jonasson et al. 2014; Jönsson et al. 2009; Juraschek et al. 2016; Keogh et al. 2008; Khodabakhshi et al. 2021; Kitabchi et al. 2013; Lewis et al. 2023; Li et al. 2022; Lin et al. 2022; Michalczyk et al. 2020; Miller et al. 2009; Mueller et al. 2010; Noakes et al. 2006; O'Brien et al. 2005; Perticone et al. 2019; Phillips et al. 2008; Pittas et al. 2006; Primo et al. 2020; Prins et al. 2023; Rad et al. 2023; Rankin and Turpyn 2007; Retterstøl et al. 2018; Ruth et al. 2013; Schiavo et al. 2022; Seshadri et al. 2004; Sharman and Volek 2004; Stoernell et al. 2008; Strath et al. 2020; Tay et al. 2008, 2014, 2015; Thomson et al. 2010; Volek et al. 2003; Wolever et al. 2008; Zainordin et al. 2021; Deluis et al. 2010). Four publications reported results based on different interventions or among various subjects and were considered separate trials (Ebbeling et al. 2022; Gower and Goss 2015; Juraschek et al. 2016; Wolever et al. 2008).

### 3.1 Study and Participant Characteristics

The main characteristics of all RCTs included in the systematic review are provided in Table 1. The trials included in this meta‐analysis were published between 2003 and 2023 with a total of 5511 adults, and more current studies were included compared to the previous systematic review (*n* = 44) (Kazeminasab et al. 2024). The age range of participants was 25–72 years, and the mean BMI at the baseline ranged from 21.8 to 48.9 kg/m^2^. Most studies recruited both males and females, nine only females (Gower and Goss 2015; Khodabakhshi et al. 2021; Kitabchi et al. 2013; O'Brien et al. 2005; Rad et al. 2023; Rankin and Turpyn 2007; Sharman and Volek 2004; Thomson et al. 2010; Volek et al. 2003) and four studies included only men (Freedland et al. 2020; Lin et al. 2022; Michalczyk et al. 2020; Prins et al. 2023). The sample size of selected trials ranged from 14 to 331 subjects, most of which were conducted in the United States (Bhanpuri et al. 2018; Cardillo et al. 2006; Dansinger et al. 2005; Davis et al. 2011; Ebbeling et al. 2022; Forsythe et al. 2008; Freedland et al. 2020; Goss et al. 2020; Gower and Goss 2015; Johnston et al. 2006; Juraschek et al. 2016; Kitabchi et al. 2013; Li et al. 2022; Lin et al. 2022; Miller et al. 2009; Mueller et al. 2010; O'Brien et al. 2005; Phillips et al. 2008; Pittas et al. 2006; Prins et al. 2023; Rankin and Turpyn 2007; Ruth et al. 2013; Seshadri et al. 2004; Sharman and Volek 2004; Stoernell et al. 2008; Strath et al. 2020; Thomson et al. 2010; Volek et al. 2003), 8 in Australia (Brinkworth et al. 2009; Brinkworth, Noakes, Keogh, et al. 2004; Brinkworth, Noakes, Parker, et al. 2004; Keogh et al. 2008; Noakes et al. 2006; Tay et al. 2008, 2014, 2015), 5 in Spain (de Luis et al. 2007; de Luis et al. 2015; de Luis et al. 2020; Primo et al. 2020; Deluis et al. 2010), 3 in Canada (Jenkins et al. 2014; Durrer et al. 2021; Wolever et al. 2008), two each in Sweden (Jonasson et al. 2014; Jönsson et al. 2009), Germany (Barbosa‐Yañez et al. 2018; Flechtner‐Mors et al. 2010), Italy (Perticone et al. 2019; Schiavo et al. 2022) and Iran (Khodabakhshi et al. 2021; Rad et al. 2023), one each in Denmark (Lewis et al. 2023), Norway (Retterstøl et al. 2018), Poland (Michalczyk et al. 2020), Indonesia (Budipramana 2020), New Zealand (Harvey et al. 2019), Malaysia (Zainordin et al. 2021), South Africa (Breukelman et al. 2021) and Turkey (Erdem et al. 2022). The time on dietary intervention varied from 2 to 156 weeks. These trials included data from participants with diverse health status; 24 studies were conducted among overweight and obese adults (Brinkworth, Noakes, Keogh, et al. 2004; Cardillo et al. 2006; de Luis et al. 2007; de Luis et al. 2015; de Luis et al. 2020; Erdem et al. 2022; Goss et al. 2020; Johnston et al. 2006; Juraschek et al. 2016; Kitabchi et al. 2013; Mueller et al. 2010; O'Brien et al. 2005; Perticone et al. 2019; Phillips et al. 2008; Pittas et al. 2006; Primo et al. 2020; Rad et al. 2023; Rankin and Turpyn 2007; Ruth et al. 2013; Schiavo et al. 2022; Seshadri et al. 2004; Sharman and Volek 2004; Thomson et al. 2010; Deluis et al. 2010), 8 in healthy subjects (Ebbeling et al. 2022; Harvey et al. 2019; Lewis et al. 2023; Michalczyk et al. 2020; Miller et al. 2009; Prins et al. 2023; Retterstøl et al. 2018; Volek et al. 2003), 12 in type 2 diabetic patients (Barbosa‐Yañez et al. 2018; Bhanpuri et al. 2018; Breukelman et al. 2021; Brinkworth et al. 2009; Davis et al. 2011; Durrer et al. 2021; Jonasson et al. 2014; Jönsson et al. 2009; Tay et al. 2014; Tay et al. 2015; Wolever et al. 2008; Zainordin et al. 2021) and nine trials involved participants with one or more cardiometabolic risk factors (Jenkins et al. 2014; Brinkworth et al. 2009; Dansinger et al. 2005; Forsythe et al. 2008; Gower and Goss 2015; Keogh et al. 2008; Li et al. 2022; Noakes et al. 2006; Stoernell et al. 2008; Tay et al. 2008) such as dyslipidemia. The study population in 4 studies included patients with various cancers including prostate (Freedland et al. 2020; Lin et al. 2022), colorectal (Budipramana 2020) and breast cancer (Khodabakhshi et al. 2021). The remaining studies were performed on patients with polycystic ovarian syndrome (Gower and Goss 2015), knee osteoarthritis (Strath et al. 2020) and metabolic syndrome (Flechtner‐Mors et al. 2010). Carbohydrate restriction thresholds were different across studies; 28 trials prescribed very LCDs daily (Barbosa‐Yañez et al. 2018; Bhanpuri et al. 2018; Breukelman et al. 2021; Brinkworth et al. 2009; Cardillo et al. 2006; Dansinger et al. 2005; Davis et al. 2011; Durrer et al. 2021; Freedland et al. 2020; Goss et al. 2020; Harvey et al. 2019; Johnston et al. 2006; Keogh et al. 2008; Khodabakhshi et al. 2021; Lin et al. 2022; Miller et al. 2009; Noakes et al. 2006; Phillips et al. 2008; Prins et al. 2023; Retterstøl et al. 2018; Ruth et al. 2013; Schiavo et al. 2022; Seshadri et al. 2004; Sharman and Volek 2004; Strath et al. 2020; Tay et al. 2008; Volek et al. 2003; Zainordin et al. 2021), LCDs (10%–26% kcal/day from carbohydrate) were used in 14 studies (Budipramana 2020; Ebbeling et al. 2022; Erdem et al. 2022; Forsythe et al. 2008; Jenkins et al. 2014; Jonasson et al. 2014; Michalczyk et al. 2020; O'Brien et al. 2005; Perticone et al. 2019; Rad et al. 2023; Rankin and Turpyn 2007; Stoernell et al. 2008; Tay et al. 2014; Tay et al. 2015) and the rest of them considered moderate LCD (Brinkworth, Noakes, Keogh, et al. 2004; Brinkworth, Noakes, Parker, et al. 2004; de Luis et al. 2007, 2015, 2020; Flechtner‐Mors et al. 2010; Gower and Goss 2015; Jönsson et al. 2009; Juraschek et al. 2016; Kitabchi et al. 2013; Lewis et al. 2023; Li et al. 2022; Mueller et al. 2010; Pittas et al. 2006; Primo et al. 2020; Thomson et al. 2010; Wolever et al. 2008; Deluis et al. 2010). Baseline CRP concentration ranged between 0.293 and 55.5 mg/L with a mean and median of 9.8 and 4.5 mg/L, respectively, in trials included in the quantitative analysis.

**TABLE 1:** Key characteristics of the included trials in this systematic review and meta‐analysis.

Author	Year	Design	Country	Participants	Sample size	Age (mean or age range), year	Diet		Duration, week
							Intervention	Control	
(Noakes et al. 2006)	2023	Cross‐over	Iran	Obese	16	NR	Hypocaloric low‐carbohydrate diet: 55% fat, 25% protein, and 20% carbohydrate content	Habitual diet: 500 kcal‐ reduced calorie diet, 20%, 15%, and 65% of total daily calories from fat, protein, and carbohydrate	6
(Follmann et al. 1992)	2023	Parallel	Denmark	T2D	64	56.2	Non‐calorie‐restricted diet: < 20 E% carbohydrates, 50–60 E% fat	Control diet: 50–60 E% carbohydrates, 20–30 E% fat and 20–25 E% protein	24
(Johnston et al. 2006)	2023	Cross‐over	Denmark	Healthy adults	56	56	Traditional (marine‐based, low‐carbohydrate) foods targeting a high fat content (> 40% of the energy intake and low carbohydrate content < 30 E%)	Western (high in imported meats and carbohydrates) targeting a high carbohydrate (55–65 E%) and moderate fat (30–35 E%) content	4
(Mueller et al. 2010)	2023	Cross‐over	USA	Healthy	10	39.9	Isocaloric diet low carbohydrate, high fat diet: < 50 g/day carbohydrate, 75%–80% fat, 15%–20% protein	Isocaloric diet High carbohydrate, low fat diet: 60%–65% carbohydrate, 20% fat, 15%–20% protein	4.42
(Cardillo et al. 2006)	2022	Parallel	USA	Relatively healthy adults	147	35	The prepared low carbohydrate diets: 20% protein, 20% carbohydrate (105 g), 21% saturated fat, 25% monounsaturated fat and 11% polyunsaturated fat	Con1: the prepared high carbohydrate diets: High carb: 20% protein, 60% carbohydrate (305 g), 7% saturated fat, 8% monounsaturated fat and 5% polyunsaturated fat. Con 2: Moderate carb: 20% protein, 40% carbohydrate (205 g), 14% saturated fat, 7% saturated fat, 16% monounsaturated fat and 9% polyunsaturated fat.	20
(Dansinger et al. 2005)	2022	Parallel	Turkey	Morbidly obese patients	30	46.6	Very low‐calorie ketogenic meal replacements incorporated 10–12 kcal/kg/day of energy and 1–1.2 g/kg of protein and 30%–40% fat	Mediterranean diet included 15%–20% protein, 45%–50% carbohydrate, and 25%–35% fat.	2
(Jonasson et al. 2014)	2022	Parallel	USA	Chronic spinal cord injury with insulin resistance or pre‐diabetes	25	42.4	Low‐carbohydrate/high‐protein diet: 30% energy from protein, 40% energy from carbohydrate.	Habitual diet	8
(Jönsson et al. 2009)	2022	Parallel	USA	Prostate cancer	45	72	Low carbohydrate arm was coached to restrict carbohydrate intake to 20 g/day	Habitual diet	26
(Ghorbani et al. 2023)	2022	Parallel	UK	Adults with a slightly elevated cardiometabolic risk	15	44.2	Ad libitum low carbohydrate high fat diet: 50 g/day carbohydrates to induce ketosis and increase fat intake, 15% protein	The high carbohydrate low fat diet: 50% carbohydrates, 15% protein, and 35% fat daily	8
(Pittas et al. 2006)	2022	Parallel	Italy	Patients with obesity and Obstructive sleep apnea syndrome	70	42	Continuous Positive Airway Pressure + Low Calorie Ketogenic Diet: 4% carbohydrates, 71% fats, and 25% proteins	Continuous positive airway pressure (these patients were not prescribed a change in eating habits)	4
(DerSimonian and Laird 1986)	2022	Parallel	Denmark	T2D	67	66.7	Carbohydrate‐reduced high‐protein diet: 30% carbohydrate, 30% protein, 40% fat	Isocaloric conventional diabetes diet: 50% carbohydrate, 17% protein, 33% fat	6
(Egger et al. 2001)	2021	Parallel	South Africa	T2D	23	NR	Low carbohydrate high fat group: a diet high in fat and not more than 50 g of carbohydrates per day	Normal daily routine	16
(Budipramana 2020)	2021	Parallel	Canada	T2D	188	58.5	Low‐carbohydrate: < 50 g carbohydrate, 35–45 g fat, and ~110–120 g protein for a total of ~850–1100 kcal	2013 Diabetes Canada (formerly the Canadian Diabetes Association) Clinical Practice Guidelines	12
(Gower and Goss 2015)	2021	Parallel	Iran	Patients with locally advanced and metastatic breast cancer	60	45	An equicaloric medium‐chain triglyceride (MCT) based ketogenic diet: 6% carbohydrate, 19% PRO, 20% MCT, and 55% from FAT	Standard diet: 55% carbohydrate (210 g), 15% protein, and 30% fat	12
(Strath et al. 2020)	2021	Parallel	Malaysia	T2D	30	57	Low carbohydrate diet (< 20 g/day intake) plus protein restriction to less than 0.8 g/kg/day and low salt diet	Standard low protein (0.8 g/kg/day) and low salt diet	12
(Thomsen et al. 2022)	2020	Parallel	Indonesia	The stage‐IV Colorectal adenocarcinoma patients	24	NR	Very low carbohydrate: 1:4 ratio	Normal diet	3
(Brinkworth, Noakes, Parker, et al. 2004)	2020	Parallel	Spain	Obese	270	49.4	Severe hypocaloric diet with high protein and low carbohydrate content: 1050 cal/day, 33% fats, 33% (86.1 g) carbohydrates, and 34% proteins, with 63.8% monounsaturated, 23.5% saturated, and 12.6% polyunsaturated fats	The standard severe hypocaloric diet: 1093 cal, 27% fats, 53% carbohydrates, and 20% proteins with 67.4% monounsaturated fats, 20.9% saturated fats, and 11.6% polyunsaturated fats	12
(de Luis et al. 2015)	2020	Parallel	USA	Patients with prostate cancer	45	72	Low‐carbohydrate diets: ≤ 20 g/day carbohydrate	Habitual diet	26
(de Luis et al. 2020)	2020	Parallel	USA	Obese	34	70.2	A very low carbohydrate diet: < 10% carbohydrate, 25% protein, > 65% fat and < 10% SFA	Low fat diet: 55% carbohydrate, 25% protein and 20% fat	8
(Juraschek et al. 2016)	2020	Parallel	Poland	Healthy	35	45.8	A low‐energy moderate‐carbohydrate diet: 32% carbohydrates, 28% proteins, and 40% fat, with 20% MUFAs and 15% of PUFAs	Mixed diet: 50% carbohydrates, 20% proteins, and 30% fat, 8%–10% SFAs, 10%–12% MUFAs, and 10% PUFAs.	4
(Hozo et al. 2005)	2020	Parallel	Australia	Obese	64	35.3	Structured supervised exercise program + low‐carbohydrate meals, not exceed in total 50 g of carbohydrate per day	Structured supervised exercise program + standard dietary advice	8
(Miller et al. 2009)	2020	Parallel	Spain	Obese	268	53.5	The low‐calorie, high protein, and low‐carbohydrate diet: 1050 cal daily with 33% fat, 33% carbohydrates, and 34% protein, with 63.8% monounsaturated, 23.5% saturated, and 12.6% polyunsaturated fats	Standard protein low‐calorie diet: 1093 cal daily, 27% fat, 53% carbohydrates, and 20% protein, with a 66.4% monounsaturated fat	39
(Ebbeling et al. 2022)	2019	Parallel	New Zealand	Healthy adults	26	38.9	Very low‐carbohydrate ketogenic diet: 5% carbohydrate	Moderate‐low carbohydrate diet: 25% carbohydrate	12
(Li et al. 2022)	2019	Parallel	Italy	Obese	50	46.8	A very low‐calorie ketogenic diet: 60%–60% protein, 20%–30% lipids, and 20% carbohydrates	The traditional Mediterranean diet: 55%–60% carbohydrates, 10%–15% proteins, and 25%–30% lipids	52.20
(Rankin and Turpyn 2007)	2020	Parallel	USA	Individuals with Knee osteoarthritis	14	70.7	The low‐carbohydrate diet: 20 g/d	The low‐fat diet: 60% carbohydrates, 20% protein, and 20% fats	12
(Higgins and Thompson 2002)	2018	Parallel	Germany	T2D	36	63	Hypocaloric very low carbohydrate: 60%–70% fat, 5%–10% carbohydrate, 20%–30% protein.	Low‐fat diet: < 30% fat, 50% carbohydrates, and 20% protein	3
(Egger et al. 1997)	2018	Parallel	USA	T2D	263	53	Continuous care intervention including nutritional ketosis	Usual care: Dietary advice according to American Diabetes Association guidelines	52
(Perticone et al. 2019)	2018	Parallel	Norway	Healthy normal weight	28	25.4	A low carbohydrate/high fat diet:< 20 g carbohydrate or no > 5% of total energy	Habitual diet ad libitum	3
(Freedland et al. 2020)	2016	Cross‐over	USA	Overweight or obese adults without diabetes	163	52.6	Low GI (GI ≤ 45) with low carbohydrate (40%), Low carbohydrate (40%) with high GI (GI ≥ 65)	Con1: High carbohydrate (58%) with low GI (GI ≤ 45), Con 2: High carbohydrate (58%) with high GI (GI ≥ 65)	5
(Brinkworth, Noakes, Keogh, et al. 2004)	2015	Parallel	Spain	Obese non‐ diabetic outpatients	331	50.1	High protein/low carbohydrate hypocaloric diet: 1050 cal/day, 33% carbohydrates, 33% fats, and 34% proteins, 23.5% SFA, 63.8% MUFA and 12.6% PUFA	The standard hypocaloric diet: 1093 cal/day, 53% carbohydrates, 27% fats, and 20% proteins, 20.9% SFA, 67.4% MUFA and 11.6% PUFA	28.5
(Schiavo et al. 2022)	2015	Parallel	Australia	Obese adults with T2D	112	58	A very‐low‐carbohydrate diet: 14% carbohydrates, 28% protein, and 58% fat, 10% SFA, 35% MUFA and 13% PUFA	A high carbohydrate, low‐fat diet with supervised aerobic and resistance exercise (60 min; 3 days/week.): 53% carbohydrates, 17% protein, and 30% fat (15% MUFA and 9% PUFA)	52
(Durrer et al. 2021)	2014	Parallel	USA	Participants with obesity‐no diabetes‐PCOS	69	35	Low carbohydrate diet: 43% carbohydrate,18% protein, and 39% fat	Lowe fat diet: 55% carbohydrate, 18% protein, and 27% fat	8
(Durrer et al. 2021)	2014	Cross‐over	USA	Women with polycystic ovary syndrome	30	31.2	Lowe carbohydrate diet: 41% carbohydrate, 19% protein, and 40% fat	Lower‐fat diet: 55% carbohydrate, 18% protein, and 27% fat	8
(Mooradian 2020)	2014	Parallel	Canada	Overweight hyperlipidemic patients	39	56.5	The low carbohydrate vegan diet: 26% carbohydrates, 31% protein, and 43% fat	High carbohydrate lacto‐ovo vegetarian diet: 58% carbohydrate,16% protein and 25% fat	26
(Flechtner‐Mors et al. 2010)	2014	Parallel	Sweden	T2D	59	62	Low‐carbohydrate diet: 20% carbohydrates	Traditional low‐fat diet: 30% fat, 49% carbohydrate	26
(Ruth et al. 2013)	2014	Parallel	Australia	Obese adults with T2D	84	58	Hypocaloric very low‐carbohydrate diet: 14% carbohydrate, 28% protein, and 58% fat, < 10% SFA, 35% MUFA and 13% PUFA	High‐unrefined carbohydrate, low‐fat diet: 53% carbohydrates, 17% protein, 30% fat, combined with structured exercise	24
(Harvey et al. 2019)	2013	Parallel	USA	Obese, premenopausal women	24	35	High protein–low carbohydrate diet: 40% carbohydrates, 30% fat, and 30% protein	High carbohydrate–low protein diet: 55% carbohydrates, 30% fat, and 15 protein	26
(Phillips et al. 2008)	2013	Parallel	USA	Obese subjects	33	42.5	High fat, low carbohydrate diet: ≤ 40 g/day carbohydrates, 60% fat (< 7% SFA), and 35% protein	Hypocaloric low fat, high carbohydrate diet: 60% complex carbohydrates, 25% SFA, and 15% protein	12
(Breukelman et al. 2021)	2011	Parallel	USA	T2D	51	54	The low‐carbohydrate diet: 2‐week phase of carbohydrate restriction of 20–25 g/day, and carbohydrate intake was increased at 5‐g increments each week	Low‐fat diet: 25% fat	26
(Khodabakhshi et al. 2021)	2011	Parallel	USA	Overweight subjects	14	47.5	Carbohydrate‐controlled weight loss diet: 30% carbohydrate, 50% fat, and 20% protein.	Fat‐controlled weight loss diet: 30% fat and 50% carbohydrate, 20% protein	20
(Tay et al. 2008)	2010	Parallel	Spain	Patients with obesity	248	43.2	Low carbohydrate diet: 1507 kcal/day, 38% carbohydrates, 26% proteins, 36% fats	Low fat diet: 1500 kcal/day, 53% carbohydrates, 20% proteins, 27% fats	13
(Davis et al. 2011)	2010	Parallel	Germany	Participants with obesity and metabolic syndrome	74	49.8	The energy‐restricted high‐protein diet: 1.34 g protein/kg body weight with 30% fat‐derived energy; 40% carbohydrates, 30% protein, and 30% fat	Energy‐restricted high‐protein diet: 40% carbohydrate, 30% protein, and 30% fat	52
(Seshadri et al. 2004)	2010	Parallel	USA	Overweight postmenopausal breast cancer survivors	40	56.2	Calorie‐restricted, modified Atkins/reduced carbohydrate diet: 35% carbohydrate, 25%–30% protein, and 35%–40% fat with greater MUFA	Calorie‐restricted, low‐fat diet: 55%–60% carbohydrates, 25% fat, 15%–20% protein	26
(Duval and Tweedie 2000)	2009	Parallel	Australia	Participants with abdominal obesity and at least one additional metabolic syndrome risk factor	60	51.4	Moderate energy restricted low carbohydrate Diet: 4% carbohydrate, 35% protein, 61% fat with the objective to restrict carbohydrate intake to, 20 g/day for the first 8 weeks and to, 40 g/day for the remainder of the study	The low‐fat diet: 46% carbohydrates, 24% protein, 30% total fat and SFA to 10 g/day and 8% of total energy	52
(Forsythe et al. 2008)	2009	Cross‐over	Sweden	T2D	13	64.5	Paleolithic diet	The diabetes diet	13
(Keogh et al. 2008)	2009	Cross‐over	USA	Healthy adults	18	30.6	High‐fat, low‐carbohydrate Atkins diet	High‐carbohydrate, low‐fat Ornish diet	4
(de Luis et al. 2007)	2008	Parallel	USA	Overweight subjects with atherogenic dyslipidemia	40	34.7	Very low carbohydrate diet: 1504 kcal: 12% carbohydrate, 59% fat, 28% protein	Low fat diet: 1478 kcal: 56% carbohydrate, 24% fat, 20% protein and 10% SFA	12
(Goss et al. 2020)	2008	Parallel	Australia	Overweight and obese subjects	99	50	Isocaloric very low carbohydrate, high saturated‐fat weight‐loss diet: 4% carbohydrate, 35% protein, 61% fat, and 20% SFA	High carbohydrate, low saturated‐fat diet: 46% carbohydrate, 24% protein, 30% fat, < 8% SFA	8
(Lin et al. 2022)	2008	Parallel	USA	Overweight and obese	20	35.5	Low carbohydrate Atkins' style diet: 20 g carbohydrates	Low fat diet: 30% fat	6
(Rad et al. 2023)	2008	Parallel	USA	Hypertriglyceridemic subjects	23	52.7	Low carbohydrate diet: 15% carbohydrate, 20% to 30% protein, and 55%–65% fat, with SFA < 10%	Low fat diet: 50%–60% carbohydrate, 15% protein and < 30% fat	8
(Retterstøl et al. 2018)	2008	Parallel	Australia	Abdominal obese subjects	88	50.7	The energy‐restricted very‐low‐carbohydrate diet: 4% carbohydrates, 35% protein, and 61% total fat (high‐unsaturated fat, and low‐saturated fat)	High‐carbohydrate, low‐fat diet: 46% carbohydrate, 24% protein, 30% total fat (8% saturated fat)	24
(Stoernell et al. 2008)	2008	Parallel	Canada	T2D	141	59	Low carbohydrate and high‐MUFA diet: 39% carbohydrate and 40% fat, GI:59.4, GL:110	Con 1: High carbohydrate and high‐GI diet: 47% carbohydrate and 31% fat, GI:63.2, GL:135. Con 2: High carbohydrate and low‐GI diet: 52% carbohydrate and 27% fat, GI:55.1, GL:131	52
(Brinkworth et al. 2009)	2007	Parallel	Spain	Obesity non‐diabetic outpatients	90	42.9	Low carbohydrate diet: 1507 kcal/day, 38% carbohydrates, 26% proteins, 36% fats	Low fat diet: 1500 kcal/day, 52% carbohydrates, 20% proteins, 27% fats	13
(O'Brien et al. 2005)	2007	Parallel	USA	Pre‐menopausal overweight women	29	39	Ad libitum low carbohydrate, high fat, high protein diet: 61% fat, 12% carbohydrate, and 29% protein	Calorie restricted high carbohydrate, low fat, low protein diet: 23% fat, 60% carbohydrate, and 18% protein	4
(Barbosa‐Yañez et al. 2018)	2006	Parallel	USA	Severely obese individuals	53	54.5	Low‐carbohydrate diet: carbohydrate < 30 g/day	Conventional diet: caloric restricted diet (reduced by 500 cal/day) and < 30% fat	156
(Erdem et al. 2022)	2006	Parallel	USA	Overweight individuals	19	37.8	Ketogenic low‐carbohydrate diet: 5% carbohydrate, 60% fat (21% saturated fat)	Nonketogenic low‐carbohydrate diet: 40% carbohydrate, 30% fat (9% saturated fat)	6
(Kitabchi et al. 2013)	2006	Parallel	Australia	Overweight and obese subjects with at least one cardiovascular risk factor	46	48.8	High saturated fat very low carbohydrate diets: 4% carbohydrate, 61% fat and 20% protein, 20% SFA	Very low‐fat diet: 70% carbohydrate, 10% fat and 20% protein, 3% SFA	12
(Michalczyk et al. 2020)	2006	Parallel	USA	Healthy overweight adults	32	34.7	Calorie‐restricted diets low (LG) glycemic diet: 40% carbohydrate, 30% protein, 30% fat	Calorie‐restricted diets high glycemic diet: 60% carbohydrate, 20% protein, 20% fat	24
(Bhanpuri et al. 2018)	2005	Parallel	USA	Overweight or obese adults with at least one of the metabolic cardiac risk factors	80	49	Atkins diet: < 20 g carbohydrate, with a gradual increase toward 50 g daily	Ornish (fat restriction) diet: a vegetarian diet containing 10% fat, 20% protein, 70% carbohydrate	52
(Lewis et al. 2023)	2005	Parallel	USA	Obese individuals	41	43.7	Very low‐carbohydrate diet: < 20 g/day carbohydrate for 2 weeks, then increased to 60 g/day after the second week	Low‐fat diet: daily calorie intake was limited to 1200 kcal	13
(Gram‐Kampmann et al. 2023)	2004	Parallel	Australia	Obese non‐dietetic subjects with hyperinsulinemia	43	50.2	High protein diet: 40% carbohydrates, 30% protein, and 30% fat	Standard protein diet: 55% carbohydrate 15% protein and 30% fat	68
(Perissiou et al. 2020)	2004	Parallel	Australia	Obese patients with T2D	38	61.8	High protein diet: 40% carbohydrates, 30% protein, and 30% fat	Low protein diet: 55% carbohydrate, 15% protein and 30% fat	64
(Primo et al. 2020)	2004	Parallel	USA	Severely obese subjects	78	54.5	Low‐carbohydrate diet: carbohydrate < 30 g/day	Conventional diet: < 30% of calories from fat and reduce caloric intake by 500 cal per day	26
(Prins et al. 2023)	2004	Cross‐over	USA	Overweight subjects	15	33.2	Hypo energetic very‐low‐carbohydrate diet: < 10% carbohydrate, 30% protein, and 60% fat	Low‐fat diet: 55% carbohydrate, 20% protein, and %25 fat	6
(Sharman and Volek 2004)	2003	Cross‐over	USA	Healthy, Normal Weight	10	26.3	Very low carbohydrate diet: < 10% carbohydrate, 30% protein, 60% fat	Low fat diet: < 30% fat: 55% carbohydrates, 20% protein, 25% fat	4

Abbreviations: GI, glycemic index; GL, glycemic load; MUFA, monounsaturated fatty acid; PUFA, polyunsaturated fatty acid; SFA, saturated fatty acid; T2D, type 2 diabetes.

### 3.2 Quality Assessment of the Included Studies

The results of quality assessment of eligible studies are shown in Table 2. The majority of studies were rated as fair (Gram‐Kampmann et al. 2023; Perissiou et al. 2020; Bhanpuri et al. 2018; Breukelman et al. 2021; Brinkworth, Noakes, Parker, et al. 2004; Budipramana 2020; de Luis et al. 2007, 2015, 2020; Erdem et al. 2022; Forsythe et al. 2008; Gower and Goss 2015; Johnston et al. 2006; Juraschek et al. 2016; Keogh et al. 2008; Khodabakhshi et al. 2021; Kitabchi et al. 2013; Lin et al. 2022; Miller et al. 2009; Mueller et al. 2010; O'Brien et al. 2005; Perticone et al. 2019; Primo et al. 2020; Prins et al. 2023; Rankin and Turpyn 2007; Retterstøl et al. 2018; Schiavo et al. 2022; Sharman and Volek 2004; Stoernell et al. 2008; Strath et al. 2020; Tay et al. 2008; Thomson et al. 2010; Volek et al. 2003; Deluis et al. 2010) whereas 22 trials were regarded as good (Jenkins et al. 2014; McCullough et al. 2022; Thomsen et al. 2022; Dansinger et al. 2005; Davis et al. 2011; Durrer et al. 2021; Ebbeling et al. 2022; Goss et al. 2020; Harvey et al. 2019; Jonasson et al. 2014; Jönsson et al. 2009; Lewis et al. 2023; Li et al. 2022; Michalczyk et al. 2020; Phillips et al. 2008; Pittas et al. 2006; Rad et al. 2023; Ruth et al. 2013; Tay et al. 2014, 2015; Wolever et al. 2008; Zainordin et al. 2021) and 8 as weak (Barbosa‐Yañez et al. 2018; Brinkworth et al. 2009; Brinkworth, Noakes, Keogh, et al. 2004; Cardillo et al. 2006; Flechtner‐Mors et al. 2010; Freedland et al. 2020; Noakes et al. 2006; Seshadri et al. 2004). Except in Lewis and Zainordin (Lewis et al. 2023; Zainordin et al. 2021), there was unclear risk of bias across some key domains in selected studies. Selection bias due to inadequate generation of a randomized sequence (Gram‐Kampmann et al. 2023; Bhanpuri et al. 2018; Retterstøl et al. 2018) and lack of allocation concealment (Bhanpuri et al. 2018; Harvey et al. 2019; Lewis et al. 2023) could not be prevented in some studies (3 trials each). Seventeen studies were susceptible to a high risk of performance bias as they did not mask participants and personnel from knowledge of intervention (Jenkins et al. 2014; Gram‐Kampmann et al. 2023; Perissiou et al. 2020; Thomsen et al. 2022; Bhanpuri et al. 2018; Budipramana 2020; Durrer et al. 2021; Goss et al. 2020; Jönsson et al. 2009; Khodabakhshi et al. 2021; Lewis et al. 2023; Michalczyk et al. 2020; Perticone et al. 2019; Pittas et al. 2006; Retterstøl et al. 2018; Tay et al. 2014; Zainordin et al. 2021). There were five studies classified to be of high risk according to detection bias due to no blinding of outcome assessors (Budipramana 2020; Durrer et al. 2021; Jönsson et al. 2009; Khodabakhshi et al. 2021; Zainordin et al. 2021). A concern about attrition bias due to incomplete result data was notable in 18 studies (McCullough et al. 2022; Perissiou et al. 2020; Barbosa‐Yañez et al. 2018; Brinkworth et al. 2009; Brinkworth, Noakes, Keogh, et al. 2004; Brinkworth, Noakes, Parker, et al. 2004; Cardillo et al. 2006; Flechtner‐Mors et al. 2010; Freedland et al. 2020; Harvey et al. 2019; Khodabakhshi et al. 2021; Kitabchi et al. 2013; Li et al. 2022; Miller et al. 2009; Rad et al. 2023; Ruth et al. 2013; Seshadri et al. 2004; Zainordin et al. 2021). Eight studies showed a high risk of reporting bias since they described selective outcomes (Gram‐Kampmann et al. 2023; Barbosa‐Yañez et al. 2018; Freedland et al. 2020; Kitabchi et al. 2013; Noakes et al. 2006; Phillips et al. 2008; Schiavo et al. 2022; Seshadri et al. 2004).

**TABLE 2:** Quality of bias assessment of the included studies according to the Cochrane guidelines.

Author name, year of publication, references	Random sequence generation	Allocation concealment	Blinding of participants and personnel	Blinding of outcome assessment	Incomplete outcome data	Selective reporting	Overall quality good/fair
Rad et al. 2023	L	L	U	U	H	L	Good
Gram‐Kampmann et al. 2023	H	U	H	L	L	H	Fair
Lewis et al. 2023	L	H	H	L	L	L	Good
Prins et al. 2023	U	U	U	U	L	L	Fair
Ebbeling et al. 2022	L	U	L	U	L	L	Good
Erdem et al. 2022	U	U	U	U	L	L	Fair
Li et al. 2022	L	L	U	L	H	L	Good
Lin et al. 2022	U	U	U	U	L	L	Fair
McCullough et al. 2022	L	L	U	U	H	L	Good
Schiavo et al. 2022	L	U	U	U	L	H	Fair
Thomsen et al. 2022	L	U	H	U	L	L	Good
Breukelman et al. 2021	U	U	U	U	L	L	Fair
Durrer et al. 2021	L	U	H	H	L	L	Good
Khodabakhshi et al. 2021	L	U	H	H	H	L	Fair
Zainordin et al. 2021	L	L	H	H	H	L	Good
Budipramana 2020	U	U	H	H	L	L	Fair
Deluis et al. 2010	U	U	U	U	L	L	Fair
Freedland et al. 2020	L	U	U	U	H	H	Weak
Goss et al. 2020	L	L	H	U	L	L	Good
Michalczyk et al. 2020	U	L	H	U	L	L	Good
Perissiou et al. 2020	U	L	H	U	H	L	Fair
Primo et al. 2020	U	U	U	U	L	L	Fair
Harvey et al. 2019	L	H	U	L	H	L	Good
Perticone et al. 2019	U	U	H	U	L	L	Fair
Strath et al. 2020	U	U	U	U	L	L	Fair
Barbosa‐Yañez et al. 2018	L	U	U	U	H	H	Weak
Bhanpuri et al. 2018	H	H	H	U	L	L	Fair
Retterstøl et al. 2018	H	U	H	U	L	L	Fair
Juraschek et al. 2016	U	U	U	U	L	L	Fair
Deluis et al. 2010	U	U	U	U	L	L	Fair
Tay et al. 2015	L	U	L	U	L	L	Good
Gower and Goss 2015	U	U	U	U	L	L	Fair
Jenkins et al. 2014	U	U	H	L	L	L	Good
Jonasson et al. 2014	L	U	U	U	L	L	Good
Tay et al. 2014	L	U	H	L	L	L	Good
Kitabchi et al. 2013	L	U	U	L	H	H	Fair
Ruth et al. 2013	L	U	U	L	H	L	Good
Davis et al. 2011	U	U	U	L	L	L	Good
Deluis et al. 2010	U	U	U	U	L	L	Fair
Mueller et al. 2010	U	U	U	U	L	L	Fair
Flechtner‐Mors et al. 2010	U	U	U	U	H	L	Weak
Thomson et al. 2010	U	U	U	U	L	L	Fair
Brinkworth et al. 2009	U	U	U	U	H	L	Weak
Jönsson et al. 2009	U	L	H	H	L	L	Good
Miller et al. 2009	U	U	U	L	H	L	Fair
Forsythe et al. 2008	U	U	U	U	L	L	Fair
Keogh et al. 2008	U	U	U	U	L	L	Fair
Phillips et al. 2008	L	L	U	L	L	H	Good
Stoernell et al. 2008	U	U	U	U	L	L	Fiar
Tay et al. 2008	U	U	U	U	L	L	Fair
Wolever et al. 2008	L	L	U	U	L	L	Good
Deluis et al. 2010	U	U	U	U	L	L	Fair
Rankin and Turpyn 2007	U	U	U	U	L	L	Fair
Cardillo et al. 2006	U	U	U	U	H	L	Weak
Johnston et al. 2006	U	U	U	U	L	L	Fair
Noakes et al. 2006	U	U	U	U	L	H	Weak
Pittas et al. 2006	L	U	H	L	L	L	Good
Dansinger et al. 2005	L	U	U	L	L	L	Good
O'Brien et al. 2005	U	U	U	U	L	L	Fair
Brinkworth, Noakes, Keogh, et al. 2004	U	U	U	U	H	L	Weak
Brinkworth, Noakes, Parker, et al. 2004	L	U	U	U	H	L	Fair
Seshadri et al. 2004	U	U	U	L	H	H	Weak
Sharman and Volek 2004	U	U	U	U	L	L	Fair
Volek et al. 2003	U	U	U	U	L	L	Fair

Abbreviations: H, high risk of bias; L, low risk of bias; U, unclear risk of bias.

### 3.3 Meta‐Analysis Results

**FIGURE 2:**
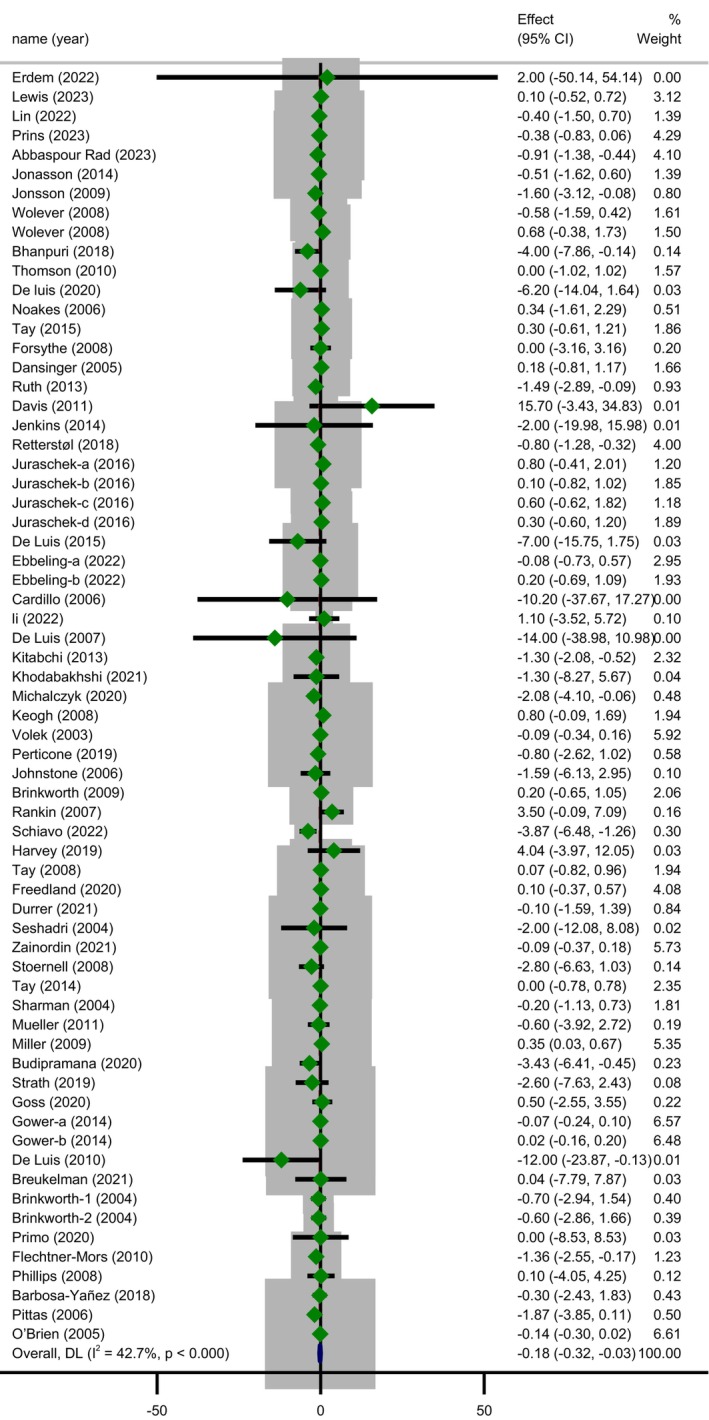
Forest plot showing the pooled effect size of low carbohydrate diets on CRP (mg/L) levels using a random effects model.

The random‐effect meta‐analysis of the results indicated that LCDs achieved an average 0.18 mg/L decrease in CRP levels compared to control groups (MD = −0.18; 95% CI: −0.32 to 0.03; *p* = 0.016) with statistically significant heterogeneity (Cochrane's *Q*‐test, *p* < 0.001, *I* ^2^ = 42.7%) (Figure 2). Sensitivity analysis by omitting each study one at a time showed the meta‐analysis result was influenced by Abbaspour Rad et al. (Rad et al. 2023) study, which changed the significance of the results (MD = −0.14; 95% CI: −0.28 to 0.00; *p* = 0.052) while the heterogeneity remained unchanged (Cochrane's *Q*‐test, *p* = 0.002, *I* ^2^ = 37.2%). The exclusion of any one trial from the analysis did not make a considerable alteration in overall effect size or heterogeneity across the included studies.

### 3.4 Subgroup and meta‐Regression Analyses

**FIGURE 3:**
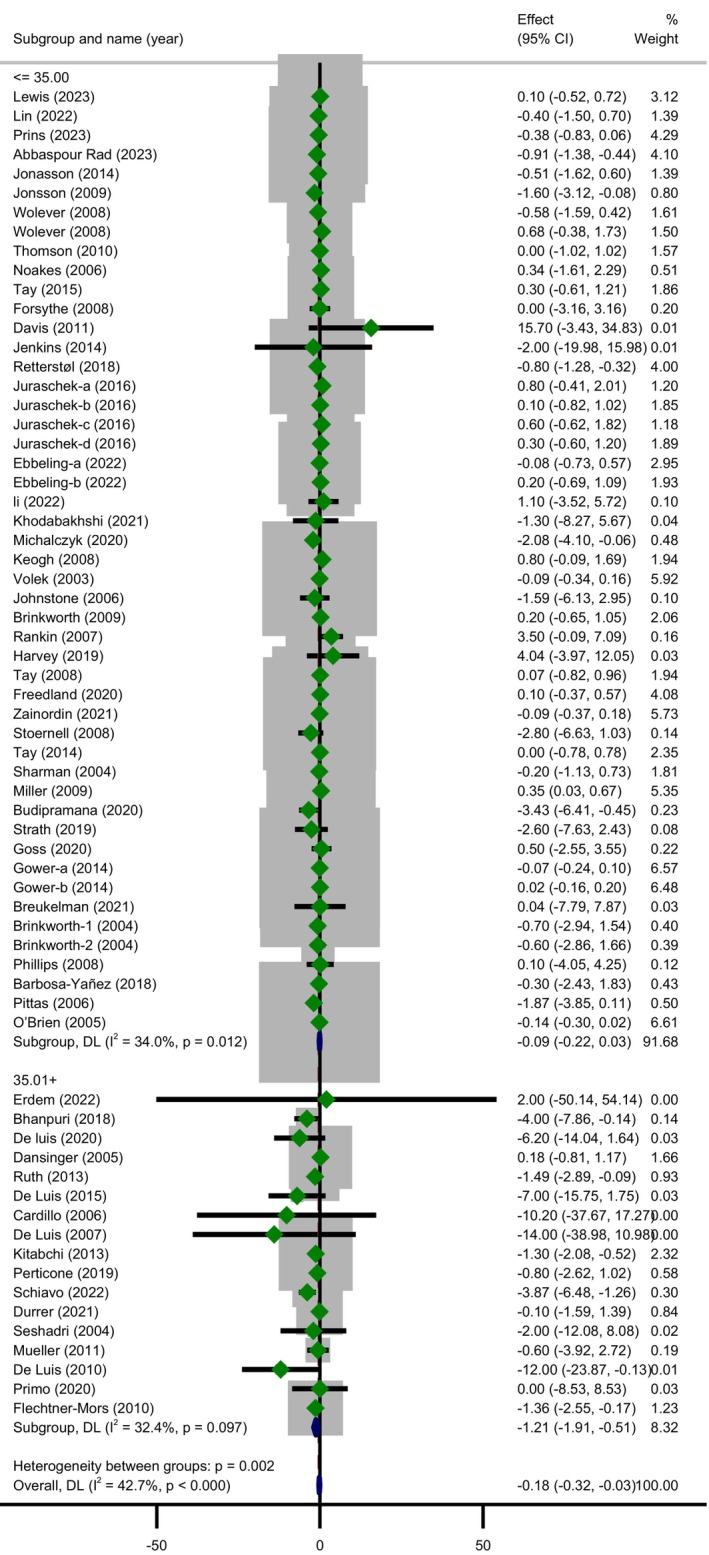
Forest plot showing the pooled effect size of low carbohydrate diets on CRP (mg/L) levels and subgroup analysis based on participants' BMI using a random effects model.

In the subgroup analysis (Table 3) based on study duration, the reduction in CRP concentration following LCDs was significant in trials with a follow‐up duration of ≥ 12.5 weeks (MD = −0.24; 95% CI: −0.48 to −0.00; *p* = 0.046) whereas the heterogeneity did not change statistically (subgroup effect, *p* = 0.526). A significant greater decline in level of CRP 0.37 mg/L (95% CI: −0.59 to −0.15; *p* = 0.001) was revealed in trials with sample size ≤ 46 with significant heterogeneity (Cochrane's *Q*‐test, *p* = 0.001, *I* ^2^ = 50.2%). However, there was a significant subgroup effect (*p* = 0.010) suggesting that this variable may be a likely source of heterogeneity. The most drop in serum CRP level was observed among subjects with baseline BMI > 35 (MD = −1.21; 95% CI: −1.91 to −0.51; *p* = 0.001) with unimportant heterogeneity (Cochrane's *Q*‐test, *p* = 0.097, *I* ^2^ = 32.4%) and this covariate (BMI) seemed to be a modifier of treatment effect (*p* = 0.002) (Figure 3). Subgroup analysis by age revealed that individuals aged 49.6 years or younger had lesser CRP concentrations than those who were older (MD = −0.30; 95% CI: −0.51 to −0.10; *p* = 0.003) with a significant moderate heterogeneity (Cochrane's *Q*‐test, *p* < 0.001, *I* ^2^ = 58.5%). It is worth to note that subgroup effect for this variable was significant (*p* = 0.041).

**TABLE 3:** Results of subgroup analyses according to intervention or participant characteristics.

Subgroup	No. of trial	Sample size (intervention/control)	Net change (95% CI)	*p*_heterogeneity_	Test of heterogeneity			
					Tau^2^ (between‐study variance)	*I* ^2^, %	*p*_within group_	*p*_between group_
Total	60	5511	−0.18 (−0.32 to −0.03)	< 0.001		42.68	0.016	
Study duration, week								0.526
≤ 12.5	29	2451	−0.14 (−0.34 to 0.05)	< 0.001	0.088	53.21	0.153	
> 12.5	31	3060	−0.24 (−0.48 to −0.00)	0.079	0.087	27.05	**0.046**	
Sample size								**0.010**
≤ 46	33	994	−0.37 (−0.59 to −0.15)	0.001	0.106	50.16	**0.001**	
> 46	27	4517	0.01 (−0.18 to 0.20)	0.089	0.046	26.01	0.906	
Proportions of carbohydrates (%)								0.691
Very LCDs (≤ 10)	28	1595	−0.11 (−0.33 to 0.11)	0.018	0.074	39.41	0.274	
LCDs (11–26)	14	798	−0.31 (−0.67 to 0.05)	0.022	0.153	47.37	0.095	
Moderate LCDs (27–44)	18	3118	−0.17 (−0.44 to 0.09)	0.013	0.090	44.12	0.187	
Age (year)								**0.040**
≤ 49.6	31	1807	−0.30 (−0.51 to −0.10)	< 0.001	0.096	58.51	**0.003**	
> 49.6	29	3704	−0.01 (−0.20 to 0.18)	0.332	0.024	8.29	0.907	
Gender								0.623
Male	4	145	−0.30 (−0.80 to 0.20)	0.144	0.106	44.56	0.252	
Female	9	326	−0.27 (−0.55 to −0.00)	0.001	0.077	69.34	**0.046**	
Both gender	48	5040	−0.13 (−0.33 to 0.08)	0.007	0.106	35.28	0.206	
Baseline BMI (kg/m^2^)								
≤ 35	43	3347	−0.09 (−0.22 to 0.03)	0.012	0.042	34.00	0.147	**0.002**
> 35	17	2164	−1.21 (−1.91 to −0.51)	0.097	0.509	32.40	**0.001**	
Baseline hs‐CRP, mg/L								
≤ 4.5	27	2882	−0.08 (−0.23 to 0.07)	0.004	0.054	44.10	0.310	**0.009**
> 4.5	33	2629	−0.70 (−1.13 to −0.26)	0.019	0.268	36.80	**0.002**	
Health status								0.348
Healthy	8	477	−0.15 (−0.46 to 0.17)	0.004	0.122	64.94	0.355	
Overweight and obese	26	3135	−0.35 (−0.64 to −0.06)	0.002	0.130	48.74	**0.018**	
T2D	12	1101	−016 (−0.51 to 0.18)	0.245	0.068	19.65	0.360	
MetS and cardiometabolic risk factors	11	624	0.05 (−0.21 to 0.31)	0.376	0.018	7.16	0.743	
Cancers	4	174	−0.46 (−1.49 to 0.57)	0.123	0.461	48.15	0.386	
Quality								0.693
Good	18	667	−0.26 (−0.58 to 0.06)	0.041	0.141	39.29	0.103	
Fair	32	3667	−0.15 (−0.34 to 0.04)	< 0.001	0.081	51.55	0.123	
Weak	10	1177	−0.07 (−0.37 to 0.24)	0.488	0.000	0.00	0.624	
Quality of fat, SFA								0.167
≤ 10%	7	514	−0.84 (−1.97 to 0.28)	0.225	0.574	26.65	0.156	
> 10%	23	3025	−0.04 (−0.20 to 0.12)	0.153	0.024	21.67	0.612	

*Note:* Bold values indicate statistically significant results (p* < 0.05).

**FIGURE 4:**
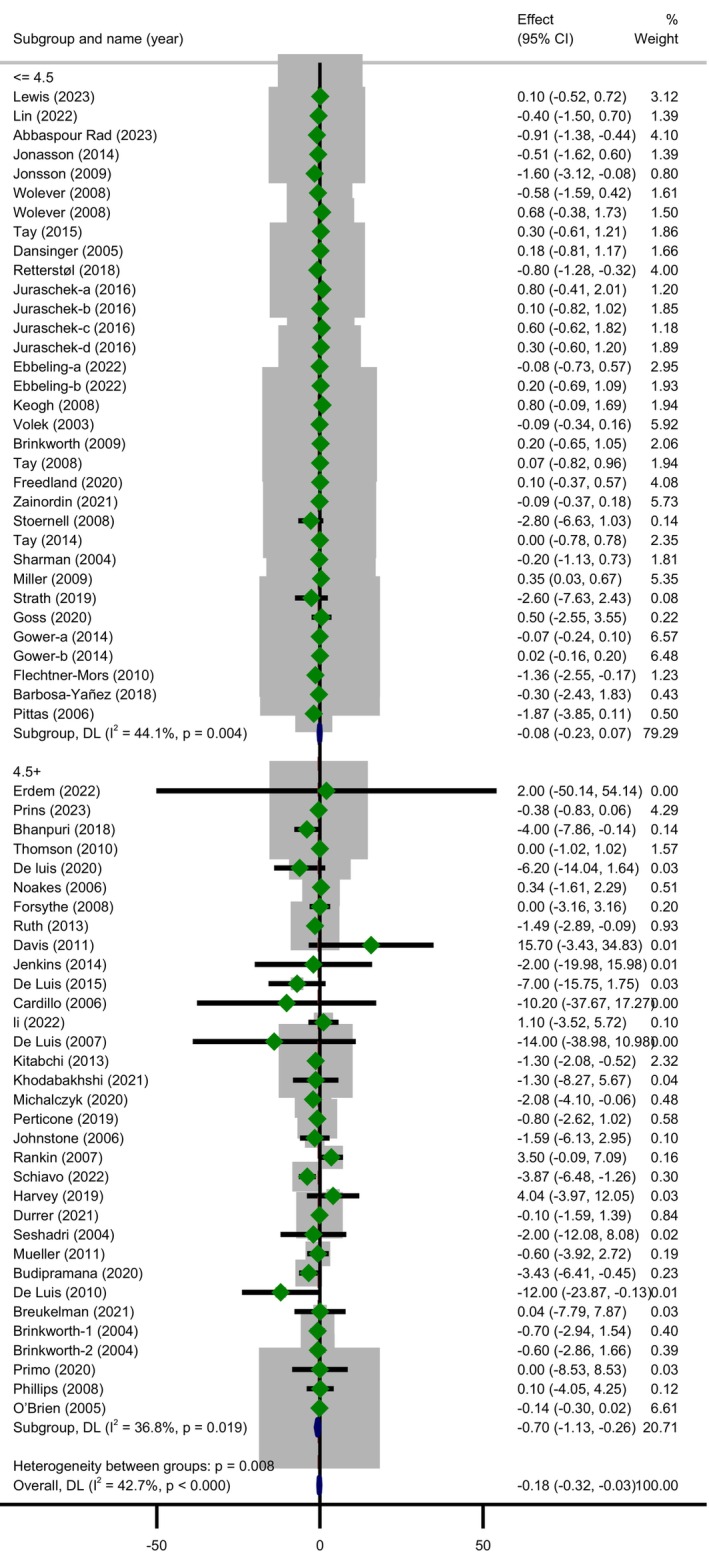
Forest plot showing the pooled effect size of low carbohydrate diets on CRP (mg/L) levels and subgroup analysis based on baseline C‐RP concentrations (≤ 4.5) using a random effects model.

Another subgroup analysis by baseline CRP concentration indicated that LCDs led to a reduction in CRP only among people with baseline CRP > 4.5 mg/L (MD =−0.70; 95% CI: −1.13 to −0.26; *p* = 0.002) and heterogeneity was not important in this subgroup (Cochrane's *Q*‐test, *p* = 0.019, *I* ^2^ = 36.8%) (Figure 4). Importantly, the effect of the LCDs on the outcome varied within these subgroups (*p* = 0.009). Other subgroup analyses showed that LCDs were able to diminish CRP only among overweight and obese people (MD =−0.35; 95% CI: −0.64 to −0.06; *p* = 0.018) with moderate heterogeneity (Cochrane's *Q*‐test, *p* = 0.002, *I* ^2^ = 48.7%) and in female subjects (MD =−0.27; 95% CI: −0.55 to −0.00; *p* = 0.046) with a relatively high significant heterogeneity (Cochrane's *Q*‐test, *p* = 0.001, *I* ^2^ = 69.3%). No statistically significant differences were identified by the rest of prespecified subgroups while it appears that the quality of included trials was a possible source of overall heterogeneity due to the disappearance of heterogeneity through some of its related subgroups (weak subgroup, Cochrane's *Q*‐test, *p* = 0.488, *I* ^2^ = 0.00%).

**FIGURE 5:**
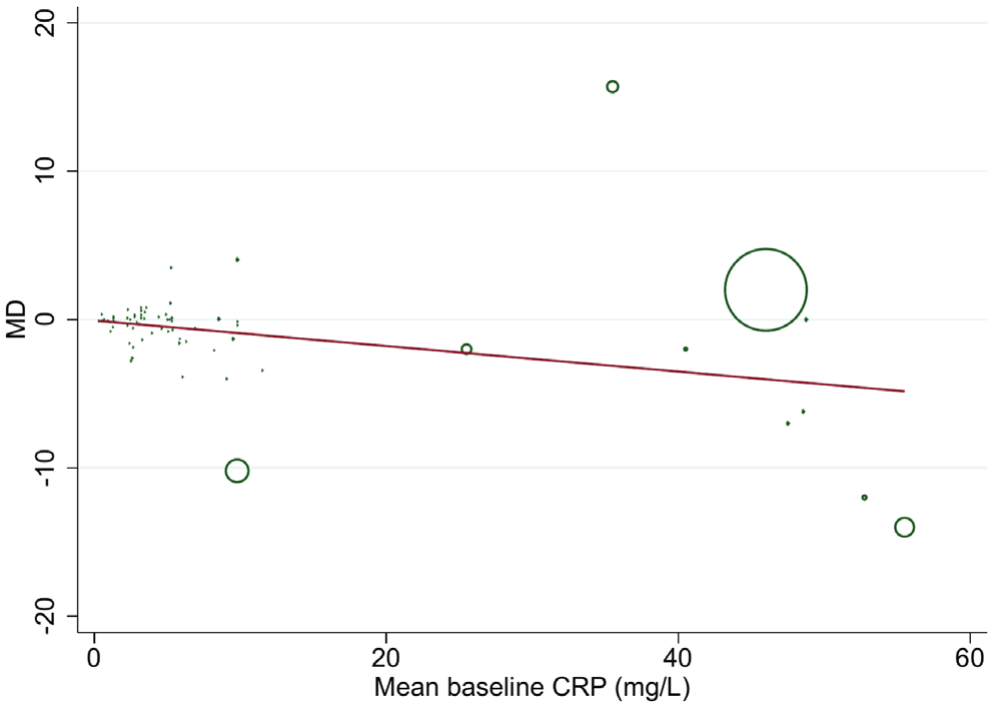
Meta‐regression plot of the effect of low carbohydrate diets on CRP concentrations. Values are in mg/L.

Univariate meta‐regression analyses revealed that none of the covariates including age (*p* = 0.320), sample size (*p* = 0.084), study duration (*p* = 0.084), the percentage of carbohydrate (*p* = 0.332) and mean of baseline BMI (*p* = 0.097), were related to the effect size. Nevertheless, baseline CRP concentration (*p* = 0.011) significantly contributed to the computed effect (Figure 5). Besides, after taking into account confounders like age, duration, and BMI, in multivariate meta‐regression analysis, baseline CRP level (*p* = 0.017) and sample size (*p* = 0.016) could significantly explain the between‐study heterogeneity.

### 3.5 Publication bias

**FIGURE 6:**
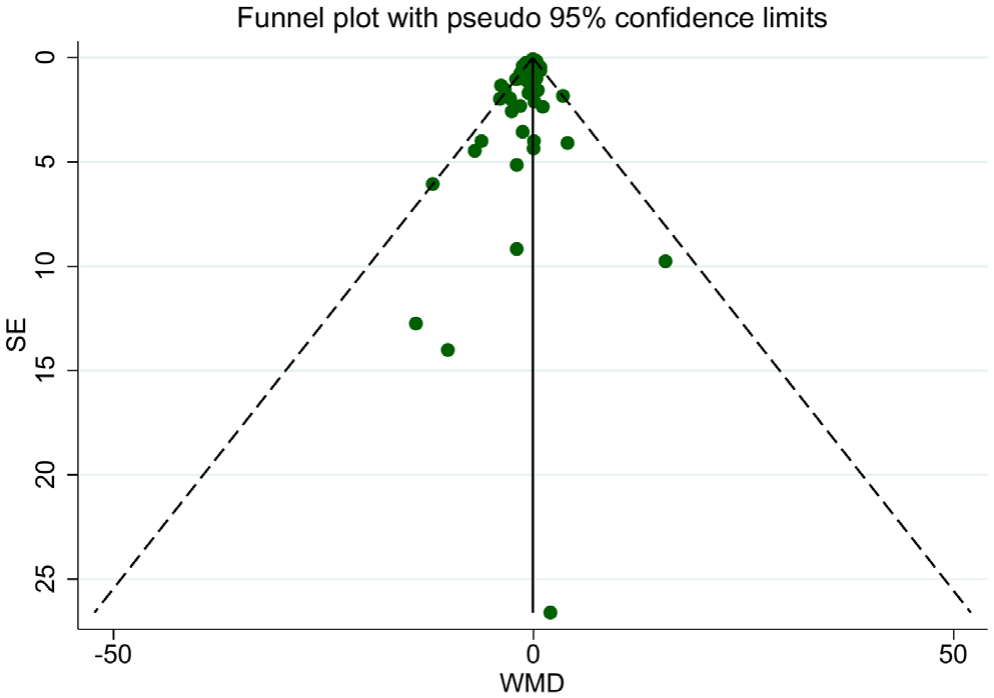
Begg's funnel plots (with pseudo 95% CIs) of the difference in means (DMs) versus the SEs of the MDs for studies that assess the influence of low carbohydrate diets on CRP.

Visual inspection of the funnel plot and the results of Egger's regression test were suggestive of significant publication bias (*p* = 0.023) (Figure 6). Usage of the Trim and fill method did not make a difference in the significance of the effect of LCDs to lower CRP concentration. Methodological heterogeneity between studies may be the source of funnel plot asymmetry.

## 4 Discussion

Given the lack of agreement on the net clinical effects of LCDs compared with control diets on inflammatory markers, the present systematic review and meta‐analysis was carried out to draw a definitive conclusion about the detrimental or beneficial impacts of this kind of diet on CRP concentration. The pooled data of 60 trials indicated subjects on LCDs achieved a greater reduction in CRP level in comparison with the control group. Nevertheless, the results of stratified analyses showed that this favorable effect was significant only in interventions of over 12.5 weeks and with a sample size ≤ 46. The lowering effects of following LCDs were greater pronounced among participants with baseline CRP concentration of more than 4.5 mg/L and those who were aged ≤ 49.6 years. Besides, a significant decrease in CRP level was seen in overweight and obese people, especially those with BMI > 30. Although subgroup analysis by gender revealed a significant decrease in the level of CRP among women, there was still a moderate between‐study variation in this category of trials. Likewise, the meta‐regression analyses supported the independent influence of baseline CRP concentration as a potential modifier of the therapeutic effects of LCDs.

Emerging scientific evidence highlights the role of diet in modulation of inflammation which contributes to the development and progression of a wide range of chronic conditions. Thus, examination of the effects of dietary approaches on blood marker of CRP, as an indicator of chronic, low‐grade inflammation is of clinical significance. A scoping review of 63 studies reporting changes in inflammatory biomarkers suggested a beneficial effect of following an LCD or a ketogenic diet in reduction of these factors (Field et al. 2023). Accordingly, a number of systematic reviews and meta‐analyses have attempted to pool the effects of LCDs on cardiovascular risk factors including CRP, and have reported inconsistent results (Santos et al. 2012; Apekey et al. 2022). Kazeminasab et al. in a recent systematic review and meta‐analysis of the influence of LCDs on inflammatory markers in adults which included 43 trials for CRP reported an important decline in mean CRP value whereas they did not stratify outcomes according to their baseline values and also type of fat alternatives to carbohydrate (Kazeminasab et al. 2024). However, they concluded that this impact might be induced by weight loss as there was a significant reduction in body weight for LCDs in comparison with LFDs. This meta‐analysis (Kazeminasab et al. 2024) included fewer trials and individuals than the present study which provided a more comprehensive literature review by including recently published articles.

Conversely, the results of a meta‐analysis of RCTs comparing the efficacy and safety of LCDs to LFDs in patients with T2DM showed no significant differences in CRP concentration (Apekey et al. 2022). Similarly, another meta‐analysis among participants with metabolic syndrome did not identify any beneficial effects for LCDs on inflammation while this type of diet had an effect on lowering insulin and body weight (Steckhan et al. 2016). The lack of consensus between the above‐mentioned meta‐analyses may be due to the heterogeneous definition of the LCD across studies. In spite of the fact that a subgroup analysis according to the proportion of carbohydrate from calorie in the intervention arm was performed in the current meta‐analysis, no statistically significant and clinically important impact on CRP was detected. Other possible reasons why LCDs had different effects on inflammatory status might be variations in dietary adherence, other dietary and lifestyle parameters, non‐modifiable factors like genetic traits, gut microbiota composition, and their interactions with diet and inflammation.

Although the overall CRP reduction seen in our meta‐analysis (~0.18 mg/L) appears modest, it aligns with reductions reported for other non‐pharmacological interventions. For instance, moderate weight loss (approximately 5%–10%) typically leads to CRP reductions of 0.5–1.5 mg/L, depending on baseline inflammatory status (Magkos et al. 2016; Selvin et al. 2007). Similarly, adherence to a Mediterranean dietary pattern has been associated with CRP decreases of about 0.3–0.9 mg/L (Schwingshackl and Hoffmann 2014; Wu et al. 2021), while regular physical activity has been shown to lower CRP by 0.26 mg/L (Fedewa et al. 2017). In contrast, pharmacological approaches such as statin therapy often produce larger reductions, typically by up to 60% (0.6–3 mg/L) (Asher and Houston 2007; Xie et al. 2024; Kandelouei et al. 2022), particularly in individuals at high cardiovascular risk (Kandelouei et al. 2022).

It is important to notice that our stratification revealed that LCDs were more possibly effective in younger people with obesity/severe obesity, results consistent with previous meta‐analyses comparing the influence of LCDs with control diets on adiponectin concentration (Ji et al. 2024). Besides, the beneficial impacts of these diets depended on the diet duration length and at least 3 months and more are required for LCDs to exert anti‐inflammatory effects. Moreover, LCDs were beneficial for improving CRP levels only among subjects with a baseline CRP > 4.5 mg/L, with additional support from meta‐regression analyses, suggesting that a higher baseline concentration may lead to a greater reduction (−0.70 mg/L) which highlights the potential of LCDs as an effective anti‐inflammatory dietary strategy for individuals with metabolic dysfunction or heightened inflammatory states. Consequently, it can be concluded that although LCDs may have only a modest impact in the general population, their clinical significance could be considerably greater in specific subgroups with elevated inflammation. This result confirmed significant declines were detected in obese/severe obese participants as low‐grade inflammation is a characteristic of the obese state (Calder et al. 2011).

Importantly, reducing dietary carbohydrates is characterized by high consumption of protein and saturated fat (Santos et al. 2012) which probably in turn induces inflammation (Fritsche 2015). On the other hand, some pieces of evidence have suggested that the effects of LCDs on health outcomes could depend on the sources of macronutrients that replaced carbohydrates (Ghorbani et al. 2023). In other words, LCDs comprised of plant‐based proteins and fats are more likely to attenuate health consequences risk than animal‐based ones (Seidelmann et al. 2018). In the present study, to address this important point of contention, we were not able to stratify the interest outcome based on the sources of fat and protein in the intervention arm, as the majority of trials had not reported any information about that. Although a subgroup analysis based on saturated fat content (%SFA ≥ 10 vs. < 10) found no significant differences in CRP levels, the lack of detailed data on macronutrient sources remains a major limitation, and further investigation into the fat and protein sources used as carbohydrate replacements is warranted.

In addition to the nature of fat, carbohydrate quality can modulate inflammation. In this regard, high glycemic diets, in particular refined carbohydrates, added sugars, sugar‐sweetened beverages, etc., appear to provoke a pro‐inflammatory response. Noteworthy, it was impossible in our work to perform a subgroup analysis by carbohydrate quality owing to variations in design and lack of reporting or incomplete data in the reviewed studies. Nonetheless, from six studies (Jenkins et al. 2014; Jönsson et al. 2009; Juraschek et al. 2016; Pittas et al. 2006; Tay et al. 2014; Wolever et al. 2008) that had reported stratified data based on the type of carbohydrate, only one (Pittas et al. 2006) revealed a favorable change in CRP following an LCD with emphasis on low glycemic index (GI) and the others did not report any changes.

Although the included randomized controlled trials did not assess mechanistic outcomes directly, several plausible biological pathways have been proposed in the broader literature to explain how LCDs may influence CRP levels. These include improvements in insulin resistance and reductions in adiposity, both of which are commonly associated with decreased systemic inflammation (Steckhan et al. 2016; Basolo et al. 2022; Shimobayashi et al. 2018). Such an improvement in insulin sensitivity may lead to an increase in glycogenolysis, gluconeogenesis, and lipolysis, especially when carbohydrate intake is less than 30 g/day (Basolo et al. 2022). Furthermore, experimental research suggests that beta‐hydroxybutyrate (BHB), the prominent metabolite of nutritional ketosis, may directly inhibit the NOD‐like receptor protein 3 (NLRP3) inflammasome and decrease the release of proinflammatory cytokines (Guo et al. 2018; Youm et al. 2015). Additionally, the appetite‐suppressing effects and reduced energy intake associated with LCDs, along with hormonal changes such as decreases in ghrelin and leptin, may support weight loss and indirectly contribute to the diminished inflammation (Puchalska and Crawford 2017; Kelly et al. 2020; Ludwig and Ebbeling 2018). Nevertheless, it is important to note that these proposed mechanisms remain theoretical within this meta‐analysis and should therefore be interpreted with caution.

The present citation analysis represents an essential update and enhancement in comparison with prior meta‐analysis (Kazeminasab et al. 2024). The major strength of this study was the RCT design of included studies, which are considered the gold standard for evaluating the causal hypotheses and, as a result, reliable conclusions can be drawn from them. Including plenty of RCTs provided an overall sample size of 5511 which allowed for maximizing the statistical power of the analyses and conducting a comprehensive subgroup analysis. Potential sources of between‐study heterogeneity were explored by subgroup and meta‐regression analyses based on some important modifiers that were not considered in previous overviews.

However, our study had some limitations that should be taken into consideration before interpreting the findings. The main limitation is related to the presence of publication bias, which could be owing to unpublished small studies with non‐significant results, leading to a lower overall certainty of the effect estimates. Although the trim‐and‐fill analysis, which detects and adjusts for this type of bias, did not change our results on treatment effect, bias may still be influencing the findings. Consequently, this decreases the certainty of our conclusions and increases the possibility that the true effect of LCDs on CRP levels may be smaller than estimated or potentially negligible. Therefore, our findings should be interpreted with caution, and future research should emphasize transparent reporting by incorporating studies with null or negative results to enhance the accuracy and reliability of the evidence regarding the true impact of LCDs on inflammation. Another key limitation of our analysis is the fragility of the observed overall effect, which was highly sensitive to one influential study (Abbaspour Rad et al.). Such dependency on one study emphasizes the need for careful interpretation of the findings and highlights the importance of replication in future trials. Besides, since most of the included publications were not a controlled feeding study and food was not provided by researchers during the study, adherence, which is crucial for the efficacy of dietary interventions, was low. In addition, diets with various macronutrient compositions were used as control diets, which makes comparison across trials difficult. For instance, some studies used low‐fat diets, while others implemented standard or mixed dietary patterns, potentially leading to significant differences in their effects on inflammatory markers like CRP. This variation in comparator diets limits the direct comparability of results and may contribute to the moderate statistical heterogeneity (*I* ^2^ = 42.7%) observed in our analysis. Therefore, varieties in the quality and macronutrient composition of control diets are a major factor that may have affected the outcomes and should be carefully considered when interpreting the pooled results. Accordingly, future research should prioritize the use of well‐defined and consistent control diets to improve comparability and reduce heterogeneity in pooled analyses.

As well as, it is necessary to point out that independent effects of LCDs might be obscured as other relevant characteristics, including macronutrient quality, lifestyle behaviors, and genetic features, have not been adjusted for within eligible studies. Besides, many of the included trials involved weight loss, which independently lowers inflammatory markers like CRP, making it difficult to attribute observed changes solely to reductions in carbohydrate intake. Furthermore, the majority of trials lacked isoenergetic designs or failed to provide detailed reporting on total energy intake, thereby limiting our capacity to fully account for caloric restriction as a potential confounder. Hence, future studies should carefully account for these confounding variables either by the design of the trial or with proper data analysis to synthesize greater robust evidence regarding the treatment effects of LCDs.

It is worth noting that although CRP serves as a useful marker of systemic inflammation, it is still considered a surrogate outcome. Thus, it is unclear whether the modest reductions observed in our meta‐analysis directly translate into meaningful reductions in long‐term clinical event rates. Accordingly, to fully elucidate the long‐term clinical relevance of low‐carbohydrate diets, future randomized trials should emphasize the assessment of hard endpoints, including cardiovascular events, type 2 diabetes incidence, and all‐cause mortality rather than relying exclusively on surrogate markers like CRP.

## 5 Conclusion

In conclusion, LCDs appear to modestly reduce CRP levels overall, with greater effects observed in trials lasting more than 12.5 weeks and among individuals with obesity and elevated baseline CRP concentrations (> 4.5 mg/L). Baseline CRP level emerged as a significant predictor of treatment response, and greater reductions were also seen among younger participants (≤ 49.6 years). These findings suggest that the anti‐inflammatory benefits of LCDs may be affected by individual characteristics, particularly baseline inflammatory status, age, and BMI. Future well‐designed randomized trials are needed to clarify the clinical relevance of these effects and to account for potential confounders such as dietary sources of fat and protein, as well as carbohydrate quality.

## Author Contributions

**Mahdieh Khodarahmi:** supervision (equal), conceptualization (equal), methodology (equal), project administration (equal), formal analysis (equal), writing – original draft (equal), writing – review and editing (equal), data curation (equal), validation (equal). **Hooria Seyedhosseini:** methodology (equal), writing – original draft (equal). **Gholamreza Askari:** conceptualization (equal), resources (equal).

## Conflicts of Interest

The authors declare no conflicts of interest.

## Supporting information


File S1.

## Data Availability

The data that support the findings of this study are available from the corresponding author upon reasonable request.
