# Correction to “Effect of Low‐Carbohydrate Diets on C‐Reactive Protein Level in Adults: A Systematic Review and Meta‐Analysis of Randomized Controlled Trials”

**DOI:** 10.1002/fsn3.71096

**Published:** 2025-10-13

Khodarahmi, M., H. Seyedhosseini‐Ghaheh, and G. Askari. 2025. “Effect of Low‐Carbohydrate Diets on C‐Reactive Protein Level in Adults: A Systematic Review and Meta‐Analysis of Randomized Controlled Trials.” *Food Science & Nutrition* 13, no. 7: e70566. https://doi.org/10.1002/fsn3.70566.

In the originally published version, the full name and affiliation for the second author were incorrect. The correct information is:

Hooria Seyedhosseini‐Ghaheh

Nutrition and Food Security Research Center, Isfahan University of Medical Sciences, Isfahan, Iran

The online version has been corrected accordingly.

We apologize for this error.

