# Supplementary material for: Effect of Low‐Carbohydrate Diets on C‐Reactive Protein Level in Adults: A Systematic Review and Meta‐Analysis of Randomized Controlled Trials
Source: Food Sci Nutr. 2025 Jul 18;13(7):e70566. doi: 10.1002/fsn3.70566 (PMC12274166; doi:10.1002/fsn3.70566)
Supplement: Supplementary file 1 — File S1. [file FSN3-13-e70566-s001.docx]

**S1 Table Search strategy in PubMed**

| **Table S1 Search strategy** |
| --- |
| **PubMed** |
| Search: ("Diet, Carbohydrate-Restricted"[Mesh] OR Carbohydrate Restricted*[Tiab] OR Diet, Carbohydrate Restricted*[Tiab] OR Diet, Low Carbohydrate*[Tiab] OR Diets, Low Carbohydrate*[Tiab] OR Carbohydrate Diet, Low*[Tiab] OR Carbohydrate Diets, Low*[Tiab] OR Low Carbohydrate*[Tiab] OR Low Carbohydrate Diet*[Tiab] OR Low Carbohydrate Diets*[Tiab] OR Carbohydrate-Restricted Diet*[Tiab] OR Carbohydrate Restricted Diet*[Tiab] OR Carbohydrate-Restricted Diets*[Tiab] OR Diets, Carbohydrate-Restricted*[Tiab] OR Carbohydrate-Restricted*[Tiab] OR Low-Carbohydrate Diet*[Tiab] OR Low-Carbohydrate*[Tiab] OR Diets, Low-Carbohydrate*[Tiab] OR "Diet, Ketogenic"[Mesh] OR Ketogenic Diet*[Tiab] OR Ketogenic*[Tiab] OR "Diet, Paleolithic"[Mesh] OR Paleolithic Diet*[Tiab] OR Paleo*[Tiab] OR Caveman*[Tiab] OR Caveman Diet*[Tiab] OR Hunter-Gatherer*[Tiab] OR Hunter Gatherer*[Tiab] OR Hunter-Gatherer Diet*[Tiab] OR atkins diet*[Tiab] OR diet, atkins*[tiab] OR modified atkins diet*[Tiab] OR MAD diet*[Tiab] OR "Diet, High-Protein Low-Carbohydrate"[Mesh] OR high-protein low-carbohydrate diet*[Tiab] OR very low carbohydrate diet*[Tiab] OR very low-carbohydrate diet*[Tiab]) AND (fasting glucose*[tiab] OR "Triglycerides"[Mesh] OR triacylglycerols*[tiab] OR "Lipoproteins, LDL"[Mesh] OR low-density lipoprotein*[tiab] OR LDL*[tiab] OR "Lipoproteins, HDL"[Mesh] OR high density lipoprotein*[tiab] OR Heavy Lipoproteins*[tiab] OR "Lipoproteins, VLDL"[Mesh] OR VLDL Lipoproteins*[tiab] OR Prebeta-Lipoproteins*[tiab] OR "insulin"[Mesh] OR fasting blood sugar*[tiab] OR "Hemoglobin A, Glycosylated"[tiab] OR HbA1c*[tiab] OR glycosylated hemoglobin*[tiab] OR "Glycated Hemoglobin A"[Mesh] OR adhesion molecules*[tiab] OR "Thrombosis"[Mesh] OR Thromboses*[tiab] OR Thrombus*[tiab] OR "cytokines"[Mesh] OR cytokine*[tiab] OR "E-Selectin"[Mesh] OR"P-Selectin"[Mesh] OR "inflammation"[Mesh] OR inflammatory markers*[Tiab] OR VCAM*[tiab] OR ICAM*[tiab] OR "selectins"[Mesh] OR "interleukins"[Mesh] OR interleukin*[tiab] OR IL6*[tiab] OR "Cell Adhesion Molecules"[Mesh] OR "Interleukin-6"[Mesh] OR IL-6*[tiab] OR Interleukin 6*[tiab] OR Tumor Necrosis Factor*[tiab] OR "Tumor Necrosis Factor-alpha"[Mesh] OR TNF alpha*[tiab] OR TNF-alpha*[tiab] OR Cachectin-Tumor Necrosis Factor*[tiab] OR TNF-alpha*[tiab] OR Tumor Necrosis Factor Ligand Superfamily Member 2*[tiab] OR Cachectin*[tiab] OR Cachectin Tumor Necrosis Factor*[tiab] OR TNF Superfamily, Member 2*[tiab] OR "Atherosclerosis"[Mesh] OR Atheroscleroses*[tiab] OR Atherogenesis*[tiab]) |
